# Repetition Rate Effects in Picosecond Laser Microprocessing of Aluminum and Steel in Water

**DOI:** 10.3390/mi8110316

**Published:** 2017-10-26

**Authors:** Ionut Nicolae, Mihaela Bojan, Cristian Viespe, Dana Miu

**Affiliations:** National Institute for Laser, Plasma and Radiation Physics, Laser Department, Atomistilor #409, 077125 Bucharest-Magurele, Romania; ionut.nicolae@inflpr.ro (I.N.); mihaela.bojan@inflpr.ro (M.B.); cristian.viespe@inflpr.ro (C.V.)

**Keywords:** picosecond laser, microprocessing, ablation in liquid, repetition rate

## Abstract

Picosecond laser drilling was studied in the case of industrial steel and aluminum, which are difficult to microprocess by conventional methods. The dependence of hole morphology and dimensions on the pulse repetition rate and number of pulses in water and air were ascertained. For both materials, the diameter of the hole is larger in water than in air. In water, the diameter is larger at higher repetition rates than at lower ones, and increases with the number of pulses. In air, the hole diameter is not affected by the repetition rate, and remains constant from 100 to 100,000 pulses. Overall, material removal is more efficient in water than in air. The shape of the hole is generally more irregular in water, becoming more so as the number of pulses is increased. This is probably due to debris being trapped in the hole, since water flowing over the target surface cannot efficiently remove it. In aluminum, the depth of the hole is smaller at higher repetition rates. By scanning the beam over the aluminum target in water, the laser penetrates a 400-μm thick workpiece, generating a line with comparable widths at the entrance and exit surfaces.

## 1. Introduction

Laser microprocessing has already proven its advantages for various materials and applications [1,2,3]. It is a clean method which, unlike mechanical or electrical discharge techniques, does not involve direct contact between the workpiece and the “tool”, which means that there is no tool wear [4,5]. Since the thermal effects of the laser beam on the target are limited, the interaction with the workpiece is highly localized, so that a high microprocessing quality can be obtained [4]. In addition, the processing of sensitive workpieces is possible, without residual stress being generated as in other methods such as electrical discharge techniques [6,7]. This is especially so in the case of short pulse lasers with femtosecond or picosecond pulse durations, where the heat-affected zone is smaller and higher processing quality is obtained than for ns pulse durations [8,9]. The high repetition rates of such short-pulse lasers lead to short microprocessing times, making them very attractive for various applications [9,10]. Lasers with picosecond pulse durations offer a combination of high precision microprocessing with limited thermal effects considered optimal for many applications, in systems that are simpler, cheaper, and more rugged than those for femtosecond lasers [10].

Ablation in a liquid environment has been shown to further increase the quality of laser microprocessing [11,12,13,14]. Water, for example, reduces the thermal damage around the processed surface by decreasing the temperature and temperature gradient [13]. In addition, the motion of the liquid carries away particulates on the surface by convection or bubble formation, even in the absence of liquid flow. The order of magnitude of ideal pulse duration for microprocessing in liquids is of several ps [15]. This is a result of the characteristic durations of various interactions between the laser pulse, target, and liquid environment [16].

The present paper presents results obtained for microprocessing in water using a laser with picosecond pulse duration, and a comparison with similar ablation in air. Laser drilling of two materials of interest in practical applications, which are difficult to microprocess using conventional methods, were studied: industrial steel and aluminum. The dependencies of the hole morphology and dimensions on repetition rate and number of pulses in the two irradiation environments were established, in order to optimize microprocessing conditions. In the case of aluminum, in addition to the stationary focusing configuration, the target was also irradiated using laser beam scanning. 

## 2. Materials and Methods 

The microprocessing experiments were conducted using a Lumera Rapid Nd-YVO_4_ laser with an 8-ps pulse duration, capable of emitting at 1.06 μm, 532 nm, and 355 nm. The 1.06-μm wavelength was used, since the threshold for plasma formation in water is lower at lower wavelengths due to higher multiphoton ionization. Above this threshold, the plasma in water leads to less efficient coupling between laser and target. The laser generates a high optical quality beam with a Gaussian profile (TEM00) with M^2^ of 1.1, which is p-polarized (>>100:1). The pulse repetition rates were varied between 10 and 500 kHz. In order to compare the effect of the pulse repetition rate on the process, an energy of 5.5 μJ/pulse was used in all cases by correspondingly modifying the average laser power for each repetition rate (the power was thus varied between 0.055 W and 2.75 W). In some cases, the targets were placed in the focus of a 30-mm focal length lens and irradiated with a stationary beam, for a different number of pulses (between 100 and 100,000 pulses). In other cases, they were scanned at 10 mm/s using an NTI type Nutfield scanner (Nutfield Technology Inc., Windham, NH, USA) with Wave Runner v 2.6 control software.

**Figure 1:**
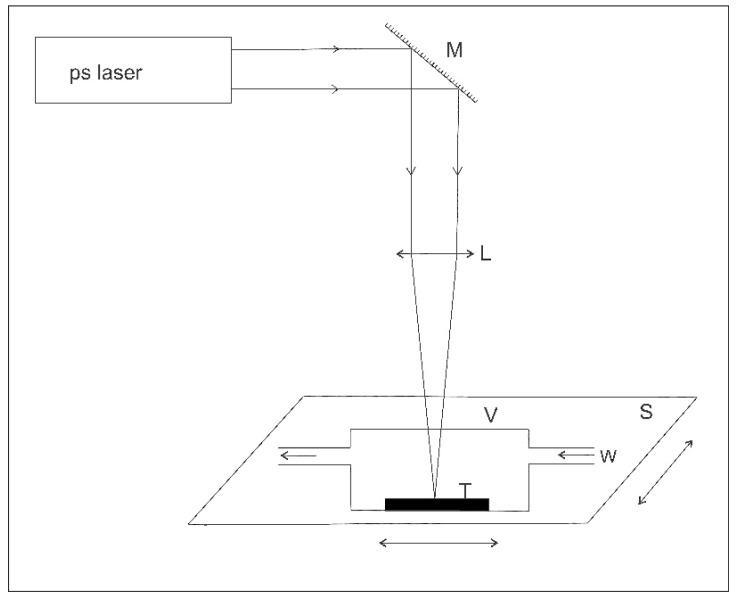
Experimental setup for stationary laser irradiation of targets in water. M—mirror; L—focusing lens; T—target; V—vessel; W—water circulated through vessel; S—x-y translation stage.

Industrial steel and aluminum targets were used for the microprocessing experiments. They were irradiated in air at atmospheric pressure or in flowing water. The ablation in water was carried out at a flow speed of 2 L/min inside a closed cylinder (height 10 mm; diameter 30 mm), which the laser beam enters through an optical window (Figure 1). Water completely fills the vessel as it flows, in order to avoid rippling at the air-water interface, which has been proven to affect laser beam propagation. The depth at which the target surface is placed in the water is 6.4 mm.

The morphology and dimensions of the resulting microstructures were analyzed using an FEI Quanta SEM (Scanning Electron Microscope) (Thermo Fisher Scientific, Waltham, MA, USA) with E = 50 keV, and an Ambios Xi-100 non-contact optical profilometer (Ambios Technology Inc., Santa Cruz, CA, USA). 

## 3. Results and Discussion

The focusing of the laser beam leads to an energy density of about 0.8 J/cm^2^, which results in a peak power density of 2 × 10^11^ W/cm^2^. This is of the same order of magnitude as the power densities routinely used when nanosecond lasers are used for microprocessing, although the energy per pulse is much lower in the case of picosecond lasers. This is due to the pulse duration of picosecond lasers being three orders of magnitude smaller. The mechanisms and timescales involved in ablation are fundamentally different for picosecond and nanosecond lasers due to this difference. In the case of picosecond pulses, the pulse durations are comparable to the timescale of energy transfer between the electrons (which absorb the incident laser energy) and the lattice. This transfer is characterized by the electron-phonon relaxation time, which is the time required for the lattice to respond to the laser pulse. The electron-phonon relaxation time is highly dependent on the material; in the case of aluminum, for example, it is 1.5 ps [17]. The energy transfer from electrons to lattice determines the rate of lattice heating, which in turn determines to what degree the relevant ablation mechanisms are thermal in nature, as well as the expansion of the ablated material [18]. Thus, the relationship between the pulse duration and the timescales of relevant ablation mechanisms is important for applications.

There are considerable differences in the processes occurring in laser ablation in air and in water. In liquids, the target species expand and a plasma is formed, just as in air, but it is more strongly confined near the target [19]. This causes a higher plasma pressure than in air for the same irradiation conditions, of the order of 10 GPa. This is accompanied by the formation of a region of high pressure in the liquid near the target, and the generation of a shock wave at times of the order of tens of ns after the laser pulse [19], which then propagates in the liquid at supersonic speeds on a μs timescale [20]. In certain cases, a cavitation bubble is formed in the liquid, presenting oscillation dynamics which persist for hundreds of μs [21]. The bubbles are formed from the vapor layer around the plasma, as energy is transferred from the plasma to the liquid. Bubble formation is observed at high laser fluences over 10 J/cm^2^ [21], which is much higher than in our case. Based on the time evolution of the processes which occur during laser ablation in liquid presented in literature [19,20,21], we can conclude that the shock wave and cavitation bubbles formed in the liquid by one laser pulse can still exist and affect irradiation by the next one when high repetition rates are used. For pulse durations of the order of several picoseconds, ultrafast boiling also occurs at the surface of the target on a picosecond timescale, leading to perturbations in the deposition of laser energy in the target [22]. In contrast, pulses longer than about 4 ps with the same energy (with the exact duration depending on the target material) are more efficient for ablation, since the heating rates do not allow the water in the target vicinity to reach its critical temperature during the duration of the pulse, so that perturbations due to ultrafast boiling do not occur [22]. The duration of the laser pulse and laser repetition rate are thus important for ablation in liquids, since they determine how much of the laser energy is used efficiently and how much is lost through shielding by various processes occurring in front of the target [19,20,21,22].

The results that we obtained in the case of the picosecond laser ablation of industrial steel show that the hole depth does not depend on repetition rate either in water or in air. We must mention the fact that there are errors in determining the depth of the holes due to target material ablated by the laser pulse which remains or is redeposited in the resulting hole, as will be discussed later. For a smaller number of pulses (500 or 1000) the depth is larger in air than in water, after which the depths become comparable. As the number of pulses increases over 5000, the depth remains constant at about 15 μm both for air and water. This indicates that there is a limiting effect that occurs in both cases, so that even after 100,000 pulses the hole depth does not increase any further.

The hole diameter behaves differently from the hole depth. In air, the diameter of the holes does not depend on the repetition rate. However, in water, the diameter depends on the laser repetition rate, as can be seen in Table 1, which compares hole dimensions after 500 pulses, at two different laser repetition rates. In the case of water, the hole diameter is larger at 250 and 500 kHz (corresponding to 4 μs and 2 μs between successive pulses, respectively) than at lower repetition rates of up to 100 kHz. This indicates a more efficient material removal at high repetition rates. The diameter of the holes also increases with the number of pulses in water. As the number of pulses increases from 500 to 100,000 pulses, the diameter increases from 80 μm to 120 μm at high repetition rates, and from about 30 μm to 80 μm at low repetition rates. The diameter for irradiation in air, on the other hand, remains constant at 30–40 μm from 100 to 100,000 pulses. Thus, after about 1000 pulses, the hole diameters are larger in water than in air for all repetition rates. Overall, the behavior of the hole diameter indicates that material removal in water is more efficient than in air. This behavior is due to the different mechanisms for material removal in air and in liquids using short-pulse lasers [16,19]. The high pressures and the shock wave generated in the liquid following the laser-target interaction may increase the efficiency of material removal [11,19].

At the moment, we have no satisfactory explanation for the dependence of hole diameter on repetition rate in the case of water. Since the change in behavior occurs between 100 and 250 kHz, which corresponds to a time between laser pulses of 10 μs and 4 μs, respectively, it could be related to the shock wave formed in the liquid at the target surface, which has a duration of the order of several μs [21].

**Figure 2:**
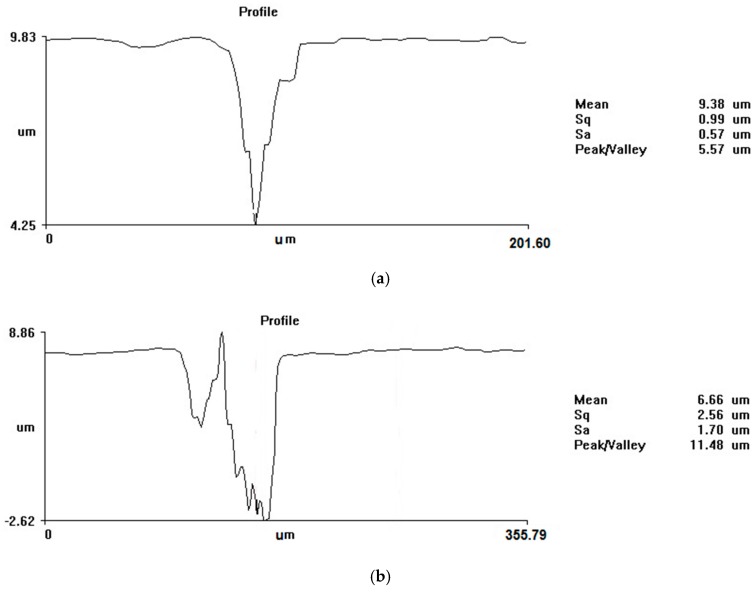
Profile of holes drilled in steel in water, using 10 kHz and (**a**) 500 pulses; (**b**) 100,000 pulses.

The shape of the hole depends on the number of pulses, becoming more irregular as the number of pulses increases (Figure 2). This hole profile indicates that a larger amount of ablated material is trapped in the hole for a larger number of pulses. The fact that the diameter of the holes increases in water with increasing number of pulses indicates that material removal at the target surface in water is more efficient than in air. For example, in Figure 2, it is shown that the diameter of the hole increased from 33 μm for 500 pulses to 80 μm for 100,000 pulses.

**Figure 3:**
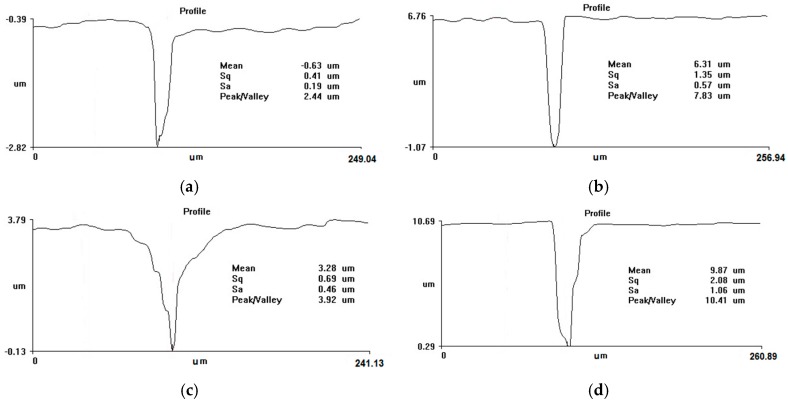

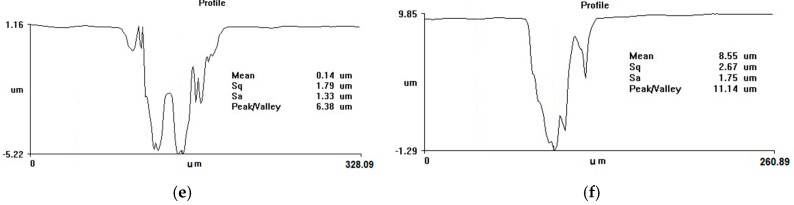
Profiles of holes drilled in aluminum in water at two different pulse repetition rates for various numbers of pulses. *(***a**) 500 kHz, 100 pulses; *(***b**) 10 kHz, 100 pulses; *(***c**) 500 kHz, 1000 pulses; *(***d**) 10 kHz, 1000 pulses; *(***e**) 500 kHz, 10,000 pulses; *(***f**) 10 kHz, 10,000 pulses.

Results obtained in the case of aluminum are similar, with some exceptions. Unlike steel, where the depth of the hole does not depend on the repetition rate, for aluminum the depth is smaller at higher repetition rates. Hole depths and diameters obtained in water at two different laser repetition rates and different numbers of ablation pulses are presented in Table 2. In aluminum, at higher repetition rates the shape of the hole is more irregular due to redeposited material, so that the depth is smaller (Figure 3). In the case of aluminum, there is a larger amount of redeposited material compared to steel. This might be due to the weaker mechanical resistance and lower melting/vaporization temperature of aluminum, which allow greater material removal by high pressure plasma and after-pulse shock-wave [23]. When the target is irradiated in water at large repetition rates, this material is confined inside the hole to a greater extent than at lower pulse repetition rates. The hole diameter, however, is, as in the case of steel, systematically larger for higher repetition rates. At small repetition rates, the holes are more regular, but the depth of the hole is still ultimately limited and does not increase after a number of pulses. 

This indicates that material removal from inside the drilled holes is the essential limiting factor in the process. This effect is more pronounced for aluminum than for steel due to the larger aspect ratio of the holes, which is evident in Figure 3. In addition, since aluminum has weaker mechanical resistance and lower melting and vaporization temperatures than steel, there is a higher density of removed material, making material redeposition more pronounced. Although laser irradiation in all of the cases we have presented takes place in flowing liquid, the flow is at the target surface and cannot efficiently remove material inside the hole. This limits the depth of the hole that can be drilled, and results in the diameter of the hole increasing with the number of pulses, whereas the depth is limited beyond a number of pulses.

All the results presented above refer to irradiation with a beam that is stationary relative to the workpiece, and drills a hole. There are several notable differences when the laser beam is scanned continuously over the target compared to the stationary beam case. In the stationary case, the beam is always incident on the same area of the target surface, so that each laser pulse impacts a surface that has been modified by the prior pulse. In addition, for stationary irradiation in water, the laser beam propagates through the liquid which contains material ablated by the preceding pulses, which absorbs part of the pulse energy. There is a flow of water maintained at the target surface both in the stationary and scanning case. However, this flow is not efficient in removing debris from inside the hole in the stationary case, since the flow is at the target surface and cannot effectively remove material inside the hole. When scanned, however, the beam moves out of the region with debris. At the same time, the formed channel allows the target material that has been ablated to be carried away efficiently by the flowing liquid. Thus, although the basic phenomena discussed in the previous paragraphs for the case of the stationary beam also appear in the case of scanning, the results are significantly different. 

**Figure 4:**
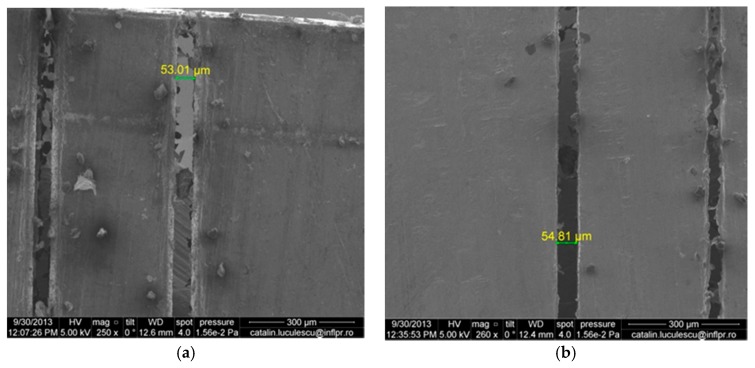
Slit obtained in a 400-μm thick Al workpiece by repeated scanning the ps beam over the target at a laser repetition rate of 500 kHz. (**a**) Entrance surface; (**b**) exit surface. The width of the slit is larger at the exit than at the entrance, contrary to laser drilling in air. The other slits in the figures are obtained with a higher scanning speed of 50 mm/s.

Figure 4 presents the results achieved when the laser beam is scanned over the aluminum target with a speed of 10 mm/s, forming a slit. A laser repetition rate of 500 kHz was used since, as discussed previously, higher repetition rates lead to greater material removal at the same number of pulses. 

As seen in Figure 4, picosecond laser processing in water has allowed us to cut completely through a 400-μm thick aluminum workpiece, obtaining a line with clean edges, without the need for high-power lasers, at a relatively low average laser power of 2.7 W. It is interesting to note that the width of the line is smaller at the entrance than at the exit of the beam (53 μm compared to 55 μm, respectively). This effect is clearly due to the presence of the liquid, since it is well known that laser drilling in air leads to a cone-shaped hole with a larger diameter at the entrance than at the exit due to the beam focusing geometry. Unlike the results obtained in many cases of drilling with a stationary beam already presented, there is a much smaller amount of debris in the hole with scanning. It is also significant that the workpiece thickness of 400 μm is much larger than the limiting depth of the holes drilled in the stationary case, of about 15 μm. This indicates that, under proper conditions, such large depths could also be obtained for holes in aluminum, if debris is removed efficiently from the processed region. Although the water is circulated over the target in both situations, only in the case of scanning is the debris efficiently removed from the hole. The scanning direction should be opposite to that of the liquid flow, so that as the beam travels in one direction across the target surface, the debris is carried away from the processed area in the opposite direction and does not interfere with the processing beam. The results presented in Figure 4 are significant since, as it is well known, aluminum is a material that is difficult to process, due to its high thermal conductivity (about 237 W/m∙K, compared to 12–45 W/m∙K for steel). Laser processing is also problematic, since aluminum has a high reflectivity and implicitly low absorption of the beam energy.

Since the laser beam is p-polarized, it is to be expected that the relative direction of polarization and scanning will affect the quality of the microprocessing. This is an aspect we intend to address in future research. We also intend to continue research by initiating studies on other metals with high thermal conductivity such as copper, gold, or silver (all of which have thermal conductivities over 300 W/m·K) for which the method could be useful. Also, increasing debris removal by controlling water flow direction and speed would be an interesting future research topic. 

## 4. Conclusions

A picosecond laser was used for comparative microprocessing experiments in water and air. Industrial steel and aluminum targets were irradiated at various laser repetition rates for a wide range of pulse numbers. For both materials, the diameter of the hole depended on repetition rate and on whether irradiation is in air or water, being larger in water than in air. In water, the diameter is larger at larger repetition rates and increases with the number of pulses. In air, the hole diameter is not affected by the repetition rate, and remains constant from 100 to 100,000 pulses. Overall, material removal is more efficient in water than in air.

The shape of the hole is irregular in water, becoming more so as the number of pulses is increased. This is due to debris being trapped in the hole, since water flowing over the target surface cannot efficiently remove it. Larger repetition rates are more efficient in material removal than lower repetition rates (at the same number of pulses) and are preferable if the material is removed from the hole. 

By scanning the laser beam (having the same energy density on the target and repetition rate as in the stationary case) over the target in corresponding conditions, a thin channel is obtained, which completely penetrates to the other side of a 400-μm thick aluminum workpiece. This thickness is considerably larger than the 15-μm deep holes otherwise obtained in the case of a stationary beam, since there are several important differences between the stationary and scanning case. 

Microtexturing of industrial grade steel parts (such as bearings) by producing regular arrays of holes like the ones presented here improves their tribological properties, decreasing their wear and increasing their lifetime. The results presented here can be used to optimize the microprocessing conditions for achieving this. Short pulse ablation in liquid has also proven to have considerable advantages for materials with high thermal conductivity, such as aluminum, which present difficulties in microprocessing.

## Figures and Tables

**Table 1:** Characteristics of holes drilled in industrial steel with 500 pulses.

Hole Dimensions	Water		Air	
	10 kHz	500 kHz	10 kHz	500 kHz
**Hole depth (μm)**	5.4	5	12	13
**Hole diameter (μm)**	33	86	32	36

**Table 2:** Characteristics of holes drilled in aluminum in water.

Hole Dimensions	10 kHz			500 kHz		
	100 p	1000 p	10,000 p	100 p	1000 p	10,000 p
**Hole depth (μm)**	8	11	11	2.4	4	6
**Hole diameter (μm)**	21	35	53	24	85	104
